# Effect of a Novel Betel Leaf Dentifrice on Commonly Seen Oral Hygiene Parameters—A Randomized Clinical Crossover Study

**DOI:** 10.3390/dj10090166

**Published:** 2022-09-06

**Authors:** Maha Ziad Ali, Wafaa Fathy Ahmed Elbaz, Saja Adouri, Vijay Desai, Salem Abu Fanas, Biju Thomas, Sudhir Rama Varma

**Affiliations:** 1 Department of Clinical Sciences, College of Dentistry, Ajman University, Ajman P.O. Box 346, United Arab Emirates; 2 Centre for Medical and Bio-Allied Health Sciences Research, Ajman University, Ajman P.O. Box 346, United Arab Emirates; 3 Department of Periodontics, AB Shetty Institute of Dental Sciences, Nitte (Deemed to be University), Mangalore 575018, India; 4 Saveetha Dental College & Hospitals, Chennai 600077, India

**Keywords:** miswak, *Salvadora persica*, betel leaf, gingivitis, dental plaque, herbal dentifrice

## Abstract

The use of herbal medicine in dentistry has grown exponentially over time. Currently, herbal medicine is considered an effective oral hygiene aid. The objective of the current study is to assess the anti-plaque efficiency and reduction of gingival bleeding of betel leaf and miswak (*Salvadora persica*) toothpaste. This randomized clinical cross-over pilot study enrolled 60 individuals with mild gingivitis. They were segregated into two groups by drawing lots. The study lasted 20 days and included a two-week washout period between miswak and betel leaf toothpaste. The gingival and plaque index were measured at specific time intervals during the research period. The results revealed that betel leaf and miswak herbal toothpaste significantly decreased plaque index. Nevertheless, betel leaf toothpaste caused a more significant reduction in gingival bleeding scores (*p* < 0.001) when compared to miswak (*p* = 0.007). No significant decrease in gingival and plaque index was seen when subjects were asked to return to their conventional chemical toothpaste. The current study concluded that betel leaf toothpaste displayed a more substantial decrease in gingival bleeding when compared to miswak toothpaste. Additionally, more studies should be done on the therapeutic benefits of betel leaf toothpaste.

## 1. Introduction

The oral cavity is ultimately the window into the general health of a patient. It is the point of interaction of the body with the external environment. Without proper oral hygiene, oral health is affected, and furthermore, the overall health and quality of life are affected [1]. The relationship between the two is many-sided and complex. Conditions and diseases such as cardiovascular disease, osteoporosis, mental health, diabetes, and stroke are well-attributed to poor oral health [2,3]. Infections, caries, and periodontal disease are all consequences of poor oral health [4]. It has been reported that a greater number of missing teeth was directly related to poor quality of life, and poor dentition affects nutritional intake [4]. Oral hygiene status has a great impact on the oral microbiome. Periodontal disease is caused by the bacteria in the biofilm and primarily affects tooth supporting structures. Gingivitis is an inflammatory condition of the soft tissue surrounding the tooth. It is caused by the formation of a layer of biofilm on the tooth surface, known as dental plaque [2]. It is made of a well-organized microbial community that in normal cases is considered balanced. However, in some cases, the balance can be disrupted, leading to dysbiotic conditions in oral health, such as bleeding gums. To ensure a continuous balance and ultimately prevent poor oral health, maintaining one’s oral hygiene regularly is necessary for removing this naturally forming layer of plaque. The consequences of poor oral health lead to dysbiosis, and a combination of cofounding factors such as age, medical conditions, medications, and low immunity can escalate the risk for periodontitis and other oral conditions [2,4].

Using a toothbrush with a dentifrice is the most common oral hygiene maintenance method [5]. For patients with limited manual dexterity and motor skills, this poses a challenge, where mouth rinses would be a better alternative [6]. However, natural methods for cleaning teeth are used in developing countries where attaining access to toothbrushes is difficult. A typical natural method is chewing sticks, which are cheap and easily accessible. Chewing sticks are made from various plants; *Salvadora persica* (miswak) is the most used plant. This plant is widespread, found in India, Iraq, Saudi Arabia, Malaysia, Sudan, Ethiopia, and southwestern Africa [5]. The favorable effects of miswak go back thousands of years ago, as it is the oldest oral hygiene tool. It is still the preferred choice of many people to this date [5]. In order to be used, branches of the tree are extracted, and the stem of the miswak stick is cut to be chewed, forming a brush that will clean food deposits stuck between teeth. This plant is rich in organic compounds such as alkaloids, phenol, organic acids, flavonoids, and inorganic compounds, such as chloride, nitrate, and sulphate [7,8]. It has deleterious actions on both aerobic and anaerobic micro-organisms, with predominant action on anaerobic bacteria and with cumulative effect on overall bacterial reduction [7]. Various studies have reported beneficial effects of miswak, such as its anti-plaque, anti-gingivitis, anti-cariogenic, promoting gingival wound healing, and whitening properties [5,7,8].

Primarily consumed in Asia, betel leaf (piper betel) is known for its many medicinal properties. It is grown throughout wide areas of southern Asia and the East Indies [9]. Green in color, the heart-shaped betel leaf gives a warm, aromatic, bitter taste. Parts of the piper betel used are the stems, roots, leaves, stalks, and fruits [9]. The numerous curative properties of the betel leaf extracts include anti-diabetic, anti-mutagenic, anti-inflammatory, antibacterial, antioxidative, and anti-hemolytic [10]. Found in high concentrations in the extracts of piper betel is sterol, which is a biologically active molecule responsible for the antibacterial property [10]. The secondary metabolites present in the piper betel plant leaves offer antibacterial properties and can be used to treat microbial infections [11]. Also present are large quantities of flavonoids and polyphenols owing to the antioxidative and anti-hemolytic activities of the extract. Another molecule found in the extracts of the betel leaf is catechol, which contains high antioxidative properties [11].

Piper betel can be used for treating alcoholism, asthma, and bronchitis. Studies have shown that the extracts from the leaves of the piper betel contain steroids, alkaloids, polyphenols, and tannins [10,11]. The leaves have a chemo-preventive potential against various conditions such as carcinoma and liver fibrosis [11]. Piper betel leaves also contain antioxidants such as eugenol, ascorbic acid, and β-carotene. The leaves are rich in nutrients, containing many vitamins and minerals, enzymes, and essential amino acids [11].

A study on chewing betel leaves showed that the saliva obtained after mastication of an entire leaf reduced the microflora by approximately 56% [12]. The piper betel is used to recover bad breath and prevent tooth decay [13]. The betel leaf has a broad-spectrum antimicrobial activity against numerous strains of bacteria, some of which include *Staphylococcus aureus*, *Bacillus cereus*, *Pseudomonas aeruginosa*, and *Escherichia coli* [13].

The current randomized control pilot study aimed to assess the efficacy of betel leaf and miswak (*Salvadora persica*) herbal toothpaste in treating gingivitis and reducing plaque.

## 2. Materials and Methods

The study was approved by the Institutional ethical committee (D-H-S-2021-NOV-24-19), performed in accordance with the Helsinki Declaration of 1975 and revised in 2013. The research was conducted from 20 October 2021 to 10 February 2022. The current study was carried out following CONSORT standards, and the study was registered on clinicaltrials.gov (NCT05363956). The following two herbal kinds of toothpaste were used in this randomized crossover clinical study: miswak toothpaste (Dabur, India), containing miswak primarily, with traces of essential oils, and betel leaf toothpaste (Bentodent, India), containing betel leaf essential oil, menthol, orange extract, and purified plant-based stevia extract with traces of natural glycolipids, grapefruit seed extract, and tree tea oil primarily. 

### 2.1. Patient Selection

Sample size estimation was performed considering an expected true difference in plaque and gingival score of 0.2 between the study groups and variance of 0.25 and considering the significance level of α = 0.05 (Z value = 1.96) and 80% power (β = 0.2, Z value = 0.84). Considering 10% loss to follow up, the final sample size was calculated as 30 per group.

n_i_ = (Z·α/2 + Z_β_)^2^·σ^2^ 2(µ_1/_µ_2_)^2^

where n denotes the sample size, Z represents the standard score, σ denotes standard deviation, and µ is the population mean of a distribution.

Subjects between the age of 20 to 60 years who are medically fit and subjects with gingival inflammation that had not progressed into periodontitis were included in the study. Participants on active treatment of antibiotics and corticosteroids who were medically compromised subjects with periodontitis (according to the AAP 2017 classification), subjects who have undergone a periodontal therapy for the past three months, and subjects who are healthy but have been using herbal products and dentifrices were excluded. 

### 2.2. Study Design

**Figure 1:**
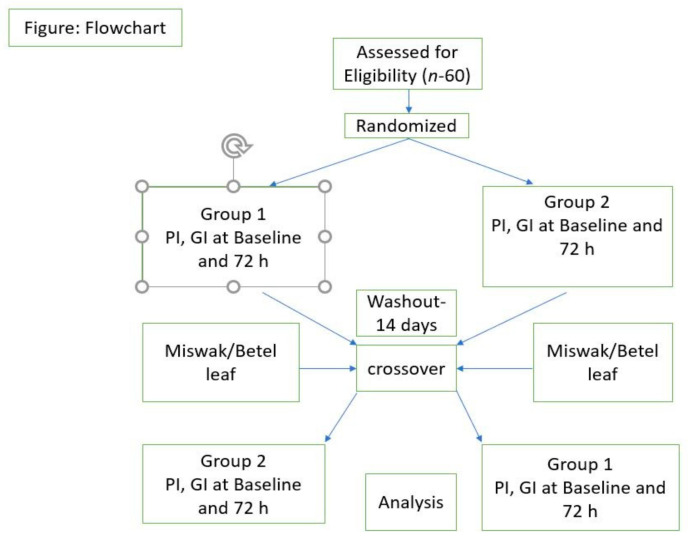
Flowchart. Selection, Randomization, and Analysis at different timepoints. PI—Plaque Index, GI—Gingival bleeding Index.

This crossover randomized single-blinded pilot study was conducted with a 20-day total examination period. The participants were informed in detail, and a signed agreement was acquired. A sample size of 60 patients with gingivitis (Score 1 in Gingival index; Loe and Sillness) was selected. Randomization was done by drawing lots into either test 1 (Dabur miswak, India) or test 2 (Betel leaf—Bentodent, India). To achieve blinding, serial opaque cardboard boxes with pre-assigned numbers corresponding to the lot randomization code were utilized to assign participants to their appropriate groups. After being assigned to their respective groups, the participants were blinded. On the first (baseline) day of the trial, all individuals were subjected to an oral examination. The baseline score was obtained for patients who met the inclusion criteria at the beginning of the study and followed-up throughout the study (Figure 1).

The participants were given a dentifrice that had been labeled and tagged with a number. Participants were asked to brush twice daily with a 1 cm line of paste in their respective brushes for two minutes, once in the morning and the other at night, using the modified bass technique. The participants were given soft toothbrushes. The technique was demonstrated to the patient, and an image of the technique was provided to the participants. The oral hygiene index (plaque index) was calculated (OHIS, Greene & Vermillion [14], and the gingival index of all teeth (Loe and Sillness [15]) were recorded at the initial appointment (baseline) in the morning. The plaque scores were recorded as “no plaque scores—0; plaque deposits found up to one-third of the tooth surface scores—1; plaque deposits found on greater than one-third but less than two-thirds of the tooth surface scores—2; plaque deposits found on greater than two-thirds of the tooth surface scores—3”. The plaque score is tabulated by adding all the individual scores and then dividing the sum by the number of teeth in which the scores were recorded. Oral examinations were evaluated in the morning and by the same examiner at all recall visits. For data entry, the toothpaste was recorded as miswak (toothpaste 1) and betel leaf (toothpaste 2).

Next, subjects were instructed to use miswak toothpaste twice a day for three days. Plaque and gingival bleeding scores were re-evaluated after 72 h. The three-day study design was developed by Marchetti et al.’s study using the 3-day plaque accumulation model [16]. Furthermore, oral prophylaxis was not instituted for the study at baseline, as this would not provide us with the efficacy of the dentifrices and was further validated by Page and Schroeder in their landmark study, where they observed the clinical presence of gingivitis by the seventh day [17].

Moreover, a 2-week washout period after using the miswak/betel leaf toothpaste was instituted to reduce the possibility of a “carry-over impact”. Patients were instructed to use any other toothpaste or any of their personal preferences during this washout period and avoid using any herbal toothpaste to prevent influencing the outcome of our study. To decrease the possibility of results bias, it is often recommended that the washout period be at least five times the treatment’s half-life [18]. The subjects were later recalled, and their plaque and gingival bleeding scores were assessed (baseline of toothpaste 2). For data entry, the toothpaste was recorded as miswak (toothpaste 1) and betel leaf (toothpaste 2). 

Betel leaf/miswak toothpaste was then provided to be used twice a day for the following three days. Gingival and plaque scores were re-measured in the morning after brushing, following 72 h of usage of toothpaste. 

### 2.3. Statistical Analysis

The raw data collected was then typed in Microsoft Excel in the form of tables. Two separate tables were made: one for control patients and the other for gingivitis patients. 

SPSS software was used for the statistical analysis (Version 28, IBM Corp, Armonk, NY, USA). The normality of the data distribution was evaluated using statistical tests. Mann–Whitney U test was used to compare the plaque and gingival index between the groups. Wilcoxon sign rank test was used to compare the plaque and gingival index values between the visit in each group. Furthermore, a *p* ≤ 0.05 was considered significant.

## 3. Results

There was no comparable statistical difference between the pastes in relation to the reduction of plaque scores (PS). Both miswak and betel leaf toothpaste provided significant efficacy in the reduction of plaque (Table 1 and Table 2). This was also evident when subjects were asked to return to using their conventional toothpaste during the washout period during the time point of the third visit (Table 2).

When PS was compared between visits, we observed a significant difference in both the toothpastes, with *p* < 0.05. We observed that from the second to fourth visit, there was a significant change in PS values in both the groups. When PS was compared between the second (after using miswak) and fourth (after using betel leaf) visit, we observed that there was no observable difference when comparing miswak (*p* = 0.001) with betel leaf (*p* = 0.001), and overall, there were no comparable significant findings in PS amongst the two pastes; both provided equal efficacy in reducing plaque (Table 1 and Table 2).

There was a significant change of (*p* < 0.001) in gingival bleeding scores between the groups (Table 3), and significance was observed when compared between each visit among the group, *p* < 0.05. Furthermore, GB values were significantly changed between the groups from baseline to the fourth visit. When evaluating the toothpaste, betel leaf showed better efficacy in reducing gingival bleeding when compared to miswak, as this was evident when comparing the timeframes between the second and fourth visit, where miswak was significantly less (0.007) when compared to betel leaf (0.001) (Table 4).

## 4. Discussion

There is a myriad of studies comparing different kinds of herbal toothpaste to each other as well as to conventional toothpaste [19,20,21]. Most of the previous research focused on the efficacy of different components in toothpaste. Moreover, herbal toothpaste contains natural ingredients, which are more acceptable because they are safer than synthetic ingredients. A study comparing the anti-plaque efficacy of miswak and tea tree oil showed significant plaque reduction among both the pastes, with slightly superior plaque reduction efficacy with miswak. The study recruited twenty-five patients. Their plaque scores were recorded, and then, they were randomly assigned to an herbal tooth paste and were asked to use it for one day and brought back for a follow up to check the plaque scores. The results of the study showed that both tea tree oil and miswak have great benefits on the reduction of plaque scores although miswak showed superior benefits to tea tree oil. The reduction from the initial plaque score was 67.3% with miswak and 59.8% with tea tree oil [4].

The results of the current study are in agreement with a previous study that compared the effect of miswak toothpaste and non-miswak toothpaste on the plaque index in 30 people with ages ranging between 18 to 26 years old, and the result showed that the reduction of plaque index was more significant among the miswak users than the non-miswak group (baseline to day 7 was 1.70/1.19 in the first group and 1.61/1.44 in the second group [22]).

In a randomized clinical trial involving herbal and non-herbal pastes that recruited fifty patients with gingivitis, they were asked to use the pastes for a period of thirty days. It was found that the herbal paste provided better anti-plaque efficacy when compared to non-herbal paste [20].

In another study, miswak had an immediate antibacterial effect against *Streptococcus mutans* but was less effective on *Lactobacilli* [23]. The ability to diminish plaque was corroborated in a previous study where it was seen that miswak interacted with the bacteria present on the tooth surface and prevented its attachment, thereby reducing plaque adhesion [24]. One of the reasons for observable reduced plaque scores was evident in our study.

Betel leaf contains essential oil that is three times more effective than fluorides. Given that the essential oils are bactericidal compared to fluorides, which are bacteriostatic, it has been further reported that it affects gingiva and aids in eliminating halitosis [25,26]. A previous study comparing betel leaf toothpaste with another non-herbal paste demonstrated the average plaque accumulation in patients having fixed orthodontic appliances and who brushed their teeth using betel leaf toothpaste was significantly less when compared to patients having the same orthodontic treatment. However, they brushed their teeth with regular toothpaste [27]. Betel leaf contains hydroxychavicol, which has an antifungal effect against many types of fungi in the oral cavity [28]. 

In a quasi-experimental study to determine if there is a difference in salivary pH before and after using betel leaf toothpaste, the data showed a significant difference, and the toothpaste was effective in increasing the salivary pH that decreases the risk of tooth decay. The mean of salivary pH was found to be 7.58 after using toothpaste with betel leaf and 7.10 before its use. This showed an average salivary pH increase of 0.48 after using betel leaf containing toothpaste. This occurs due to the creation of an alkaline environment inhibiting the plaque-forming bacteria such as *Streptococcus mutans*, which in turn prevents tooth decay. These results verify the beneficial effects that betel leaf has in oral hygiene and its role as an anti-plaque and anti-caries agent [29].

In an in vitro study evaluating the antibacterial action of betel leaf extract with miswak extract, the diameter of the inhibition zone of these herbal extracts against the bacteria was checked. The effectiveness of red betel leaf was evaluated in comparison to miswak’s due to the difference in the concentration of the extract contents. They concluded that the extracts of red betel leaf are 50% better than the extracts of miswak at inhibiting *Staphylococcus aureus* growth. This was also evident from our study, where betel leaf exhibited statistically significant gingivitis reduction compared to miswak but provided an almost equal plaque-reduction efficiency compared to miswak. This could also be attributed to the anti-hemolytic effect seen in betel leaf extracts, as reported in a study [9] that resulted in observably low gingival bleeding scores compared to miswak. 

Though betel leaf extract provides promising results, some studies showed the disadvantages of betel leaf toothpaste during the experiments. It is found that betel leaf extract gel (15%, 25%, and 35%) applied for 1, 3, or 6 months can lead to enamel surface roughness, regardless of different concentrations [30,31]. In a randomized triple-blinded clinical cross-over study, the investigators used a self-prepared herbal paste and a conventional paste to examine the effectiveness of both the pastes in reducing gingival bleeding index scores, they found that the self-prepared herbal pastes provided lesser efficacy in reducing gingival bleeding scores when compared to conventional pastes [19]. To compare the betel leaf toothpaste with the non-betel leaf contains toothpaste on the plaque accumulation, a previous study demonstrated that the average plaque accumulation in the first group (patients who have fixed orthodontic appliances and brushed their teeth by using betel leaf toothpaste) was 25.54 and 41.09 in the second group (patients who had the same orthodontic treatment but brushed their teeth with regular toothpaste) [27]. A study proposing a domiciliary protocol for oral hygiene was conducted with a six-month follow up using clinical and microbiological parameters using para-probiotics to assess plaque scores. It was found to have a significant effect [32]. The concept of polyherbal mouthwashes has lately been introduced among the vast repertoire of oral hygiene products. Some of the studies using polyherbal rinses have had positive outcomes for the treatment of dental caries; they have reported inhibitory action [33,34]. 

Our study has some limitations, such as small sample size, being a pilot study, and having a short study duration. In addition, the baseline results of group I differed considerably from group II. One reason for this is the groups were a mixture of university staff and allied medical personnel, and group 2 was comprised of mostly allied medical personnel; though lots were taken for randomization, most of these staff were novice with regards to dental hygiene maintenance, and as a result, plaque and debris scores were seen that contributed to the gingivitis, which was reflected at baseline. We also did not use control groups to determine overall efficacy, as it would be a challenge since our focus was on how plaque and gingival scores were affected in gingivitis patients. The proposal to use active controls and have a larger sample size could bring more concrete findings. Furthermore, some participants complained of enamel roughness and discoloration after using betel herbal toothpaste. Moreover, two participants given betel toothpaste reported a pungent, unpleasant taste. Betel leaf’s unpleasant taste could be considered a limitation to the present study, and determination of whether subjects used the herbal toothpaste provided to them was based solely on their responses. Nevertheless, from the present study, betel leaf shows promise and could be considered as a potential adjunct along with non-surgical periodontal therapy. 

## 5. Conclusions

Both herbal toothpastes demonstrated a significant role in the reduction of plaque and bleeding scores. Though much research was published on the effects of miswak on the treatment of gingivitis and oral hygiene maintenance, very few studies highlighted the effects of betel leaf on different parameters of oral hygiene in general. Further research on the effect of betel leaf is needed.

## Figures and Tables

**Table 1:** Comparison of Plaque Index between the groups at each visit.

	Group I		Group II		*p*-Value
	Median	IQR	Median	IQR	
Base line After miswak (72 h) Wash out—14 days After betel leaf (72 h)	0.83	0.67–1	2.5	2.16–2.66	<0.001 *
	0.33	0.16–0.5	1.5	1.45–1.8	<0.001 *
	0.67	0.5–0.87	2	1.8–2.3	<0.001 *
	0.5	0.33–0.67	1.83	1.75–2	<0.001 *

IQR, interquartile range. * *p* < 0.05, statistically significant.

**Table 2:** Comparison of Plaque Index between the time points in each group.

	Toothpaste 1 (Miswak)	Toothpaste 2 (Betel Leaf)
First–Second	<0.001 *	<0.001 *
Second–Third	<0.001 *	<0.001 *
Third–Fourth	<0.001 *	0.009 *
Second–Fourth	<0.001	<0.001

* *p* < 0.05, statistically significant.

**Table 3:** Comparison of Bleeding score between the groups at each visit.

	Group I		Group II		*p*-Value
	Median	IQR	Median	IQR	
Base line	1	0–1	3	2–3	<0.001 *
After miswak (72 h)	1	0–0	2	1–2	<0.001 *
Wash out—14 days	1	0–1	2	1–2	<0.001 *
After betel leaf (72 h)	0	0–1	1	1–2	<0.001 *

IQR, interquartile range. * *p* < 0.05, statistically significant.

**Table 4:** Comparison of bleeding score between the time points in each group.

	Toothpaste 1 (Miswak)	Toothpaste 2 (Betel Leaf)
First–Second	<0.001 *	<0.001 *
Second–Third	<0.001 *	<0.001 *
Third–Fourth	0.317	0.034 *
Second–Fourth	0.007 *	<0.001 *

* *p* < 0.05, statistically significant.

## Data Availability

The study is registered on clinicaltrials.gov (NCT05363956) and all data can be accessed.
